# α-Mangostin Enhances Betulinic Acid Cytotoxicity and Inhibits Cisplatin Cytotoxicity on HCT 116 Colorectal Carcinoma Cells

**DOI:** 10.3390/molecules17032939

**Published:** 2012-03-08

**Authors:** Abdalrahim F. A. Aisha, Khalid M. Abu-Salah, Zhari Ismail, Amin Malik Shah Abdul Majid

**Affiliations:** 1 School of Pharmaceutical Sciences, Universiti Sains Malaysia (USM), Minden 11800, Pulau Pinang, Malaysia; 2 Biochemistry Department and King Abdullah Institute for Nanotechnology, King Saud University (KSU), Riyadh 11451, Saudi Arabia

**Keywords:** α-mangostin, betulinic acid, cisplatin, apoptosis, cytotoxicity enhancement, cytoprotection effects

## Abstract

Despite the progress in colon cancer treatment, relapse is still a major obstacle. Hence, new drugs or drug combinations are required in the battle against colon cancer. α-Mangostin and betulinic acid (BA) are cytotoxic compounds that work by inducing the mitochondrial apoptosis pathway, and cisplatin is one of the most potent broad spectrum anti-tumor agents. This study aims to investigate the enhancement of BA cytotoxicity by α-mangostin, and the cytoprotection effect of α-mangostin and BA on cisplatin-induced cytotoxicity on HCT 116 human colorectal carcinoma cells. Cytotoxicity was investigated by the XTT cell proliferation test, and the apoptotic effects were investigated on early and late markers including caspases-3/7, mitochondrial membrane potential, cytoplasmic shrinkage, and chromatin condensation. The effect of α-mangostin on four signalling pathways was also investigated by the luciferase assay. α-Mangostin and BA were more cytotoxic to the colon cancer cells than to the normal colonic cells, and both compounds showed a cytoprotective effect against cisplatin-induced cytotoxicity. On the other hand, α-mangostin enhanced the cytotoxic and apoptotic effects of BA. Combination therapy hits multiple targets, which may improve the overall response to the treatment, and may reduce the likelihood of developing drug resistance by the tumor cells. Therefore, α-mangostin and BA may provide a novel combination for the treatment of colorectal carcinoma. The cytoprotective effect of the compounds against cisplatin-induced cytotoxicity may find applications as chemopreventive agents against carcinogens, irradiation and oxidative stress, or to neutralize cisplatin side effects.

## 1. Introduction

Colon cancer accounts for almost 10% of the total cases of cancer, and 8% of the total cancer deaths [1]. The first line treatment of colorectal carcinoma is based on different combinations of 5-fluorouracil, oxaliplatin, irinotecan, leucovorin and capecitabine. These combinations showed better therapeutic outcome than the corresponding monotherapies [2]. Despite the progress in colon cancer treatment, no curative therapy is available, and the available treatment is meant to prolong the disease-free interval. Therefore, there is continuous need for new drugs or drug combinations for the treatment of colorectal carcinoma.

**Figure 1:**
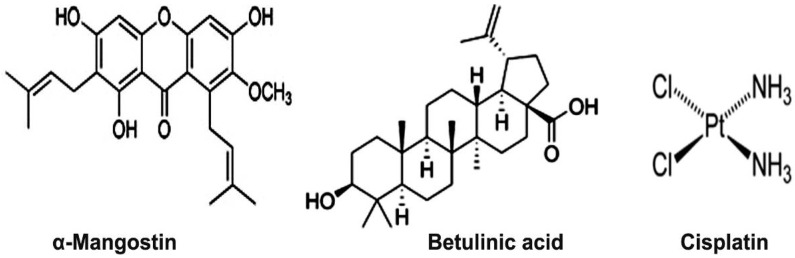
Structure of α-mangostin, betulinic acid, and cisplatin.

1,3,6-Trihydroxy-7-methoxy-2,8-bis(3-methyl-2-butenyl)-9H-xanthen-9-one (α-mangostin, Figure 1), is a heterocyclic oxygenated xanthone isolated from *Garcinia mangostana* fruit rinds. The compound has been reported to possess multiple pharmacological effects, including antioxidant, anti-inflammatory, and anti-cancer properties [3]. The anti-cancer effects of the compound are mediated via the mitochondrial apoptosis pathway [4,5].

3β-Hydroxy-20(29)-lupaene-28-oic acid (BA, Figure 1), is a pentacyclic triterpene with multiple pharmacological effects against angiogenesis, neoplasms, and inflammation [6,7,8,9,10,11]. BA was believed to be a selective anti-melanoma agent; however several studies have shown a broader range of anti-cancer activity, including prostate cancer [12], and colon cancer [13]. Previous studies showed that the anti-cancer properties of BA are also mediated via the mitochondrial apoptotic pathway [14,15].

*Cis*-diamminedichloroplatinum(II) (cisplatin, Figure 1), is one of the most potent anti-tumor agents known, and displays a broad spectrum anti-tumor activity [16]. Its cytotoxicity is mediated by interaction with DNA to form DNA adducts, which activate several signal transduction pathways leading to activation of apoptosis [16,17]. However, resistance to cisplatin after an initial response [16], and the survival and expansion of highly tumorigenic side-population cells [18] are major obstacles in cisplatin chemotherapy. Several mechanisms of cisplatin resistance have been reported, including its reduced intracellular accumulation, increased inactivation by thiol-containing molecules, increase in DNA damage repair and DNA damage tolerance, overexpression of apoptosis inhibitors such as survivin, and the presence of deregulated cell signaling pathways [16]. Another obstacle that limits its therapeutic use is the severe side effects such as nephrotoxicity, neurotoxicity, and ototoxicity. The severity of these side effects may be reduced by the combination of cisplatin with compounds with cytoprotective properties such as α-mangostin and BA. Previous studies showed that pre-treatment with the cytoprotective agent amifostine is associated with a low toxicity of cyclophosphamide, doxorubicin, vincristine and prednisone in elderly patients with aggressive non-Hodgkin’s lymphoma, without impairment of the treatment outcomes [19].

Several strategies have been tried in order to overcome the multidrug resistance in cancer chemotherapy, which include combination with inhibitors of the ATP-binding cassette transporters [20], the use of nanocarrier drug delivery systems [21,22], and drug combinations [2].

Compounds that work directly on the mitochondria may evade resistance to conventional chemotherapeutics, consequently BA and α-mangostin may hold a great promise as a novel approach to overcome drug resistance in cancer chemotherapy [15]. In this context, this study was undertaken in order to study the cytotoxic effect of α-mangostin, BA and cisplatin combinations on colorectal carcinoma cells.

## 2. Results

### 2.1. Phytochemical Analysis

**Figure 2:**
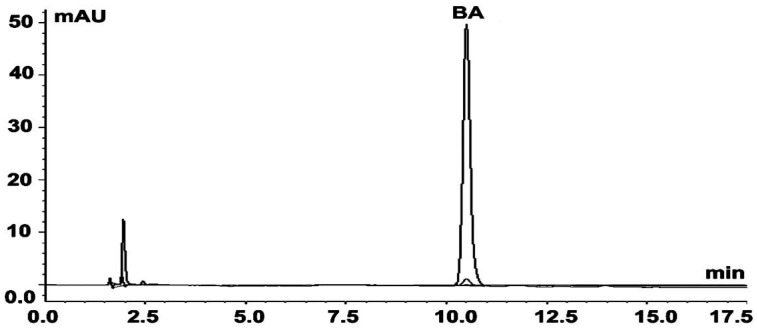
HPLC chromatogram of the isolated BA. Data acquisition was performed at 210, 230 and 254 nm.

BA was isolated from a BA fraction at 10% yield (wt/wt). The purity of the compound was determined by HPLC and was found to be >97% (Figure 2). The retention time of the isolated and reference compound was 10.62 ± 0.09 min, and 10.54 ± 0.12 min, respectively. The identity of BA was further confirmed by mass spectrometry (MS) analysis. The isotopic pattern of the isolated compound was *m/z* 455.35, 456.35 and 457.35, and was identical to that of the reference compound.

**Figure 3:**
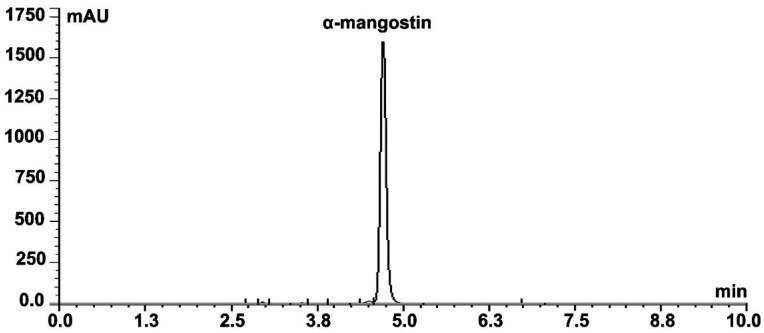
HPLC chromatogram of the isolated α-mangostin. Data collection was carried out at 244, 254, 316 and 320 nm.

α-Mangostin was isolated at 60% yield (wt/wt) from the toluene extract of *Garcinia mangostana*. The compound’s purity was tested by HPLC, which was >97% (Figure 3). The identity of the compound was determined firstly by comparing its HPLC retention time with that of the reference compound, which was 4.72 ± 0.01 min. MS analysis of the isolated α-mangostin showed identical isotopic pattern (*m/z* 409.15, 410.15 and 411.15) with the reference compound, which further confirms its identity. 

### 2.2. Cytotoxicity

**Figure 4:**
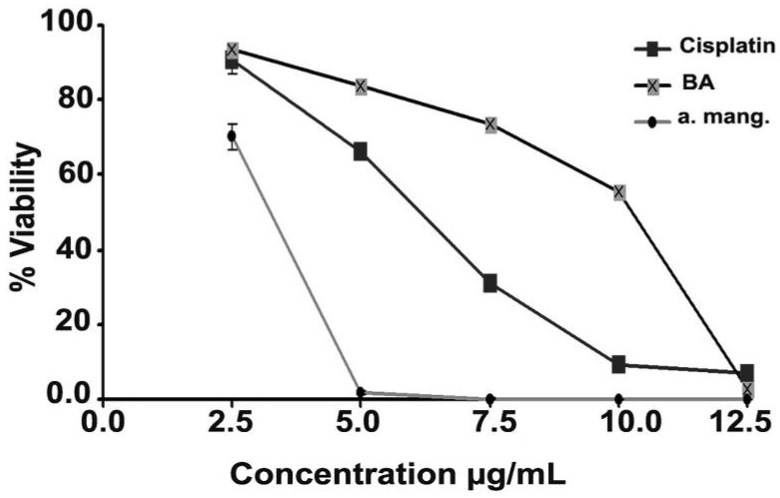
The dose response curves of α-mangostin, BA, and cisplatin on HCT 116 cells.

α-Mangostin, BA, and cisplatin inhibited the growth of HCT 116 cells in a dose dependent manner (Figure 4). The median inhibitory concentrations (IC_50s_) were calculated from the dose response curves, and were found to be 4.0 ± 1.0 µg/mL, 8.9 ± 0.1 µg/mL, and 6.5 ± 0.1 µg/mL, respectively. α-Mangostin and BA showed a lower cytotoxic effect on the normal colonic cells (CCD-18Co) compared to their effect towards the cancer cells, and the IC_50s_ were 11 ± 0.5 µg/mL and 47 ± 1.0 µg/mL, respectively.

### 2.3. Combination of α-Mangostin, BA and Cisplatin

The cytotoxicity of α-mangostin/BA, α-mangostin/cisplatin, and BA/cisplatin drug pairs was investigated. Firstly, cells were treated simultaneously with the treatment compounds at inactive concentration of the first drug and various concentrations of the second. Secondly, cells were treated with cytotoxic concentration of the first drug with various concentrations of the other compound. The percentage inhibition was determined and the drug enhancement was determined by calculating the fold change in the cytotoxicity of the combination relative to their additive effect, the results are summarized in Table 1. The results showed that α-mangostin at 2.5 µg/mL significantly enhanced the cytotoxicity of BA. In the same manner, combination of cytotoxic concentrations of α-mangostin (>5 µg/mL) with BA did not reduce the BA cytotoxicity, but showed almost 100% killing of cells in all studied combinations. On the contrary, α-mangostin at a concentration of 2.5 µg/mL significantly reduced the cisplatin cytotoxicity. It is noteworthy to mention that addition of α-mangostin at cytotoxic concentration (>5 µg/mL) did not reduce the cisplatin cytotoxicity, but caused 100% growth inhibition. Similar results were also obtained in the BA/cisplatin combinations: BA at sub-cytotoxic concentration (2.5 µg/mL) caused significant reduction in cisplatin cytotoxicity. On the contrary, BA at >7.5 µg/mL with cisplatin at different concentrations resulted in 100% killing of the colorectal carcinoma cells.

On the other hand, the α-mangostin/BA combinations did not enhance the BA cytotoxicity towards the normal colonic CCD-18Co cells. The fold change in BA cytotoxicity, relative to the additive effect of both compounds, in the following α-mangostin/BA combinations 2.5/2.5, 2.5/5.0, 2.5/7.5 and 2.5/10 µg/mL was 0.9 ± 0.1, 1.1 ± 0.1, 1.1 ± 0.1, and 1.0 ± 0.2, respectively. 

### 2.4. Effect on Caspases-3/7

**Figure 5:**
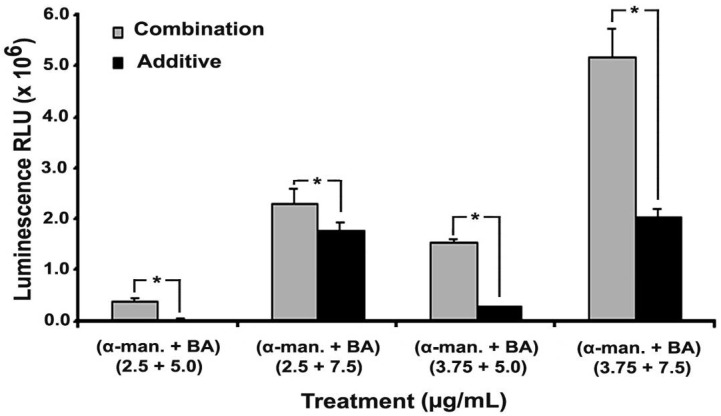
Effect of α-mangostin/BA combination on caspases 3/7 of HCT 116 cells. α-Mangostin, at 2.5 and 5.0 µg/mL, was added to betulinic acid (BA) at 5.0 and 7.5 µg/mL. The results are displayed as average relative light units (RLU). The effect of α-mangostin/BA combination was compared to the additive effect by student’s *t*-test, and (*) indicates *P* value < 0.05.

The effect of α-mangostin/BA combinations was then investigated on caspases-3/7 of HCT 116 cells. α-Mangostin at 2.5 and 3.75 µg/mL did not show significant induction of the caspases 3/7 activity compared to the negative control, *P* = 0.99 and 0.96, respectively. Likewise, BA at 5 µg/mL did not induce significant changes in caspases 3/7 activity, *P* = 0.36. On the contrary, BA at 7.5 µg/mL significantly induced the caspases activity, *P* = 0.0 (Figure 5). The effect of α-mangostin on BA-induced caspases 3/7 activity was then studied. The activity of the combination was compared to the additive effect. α-Mangostin at 2.5 and 3.75 µg/mL significantly induced the apoptotic effect of BA at 5 and 7.5 µg/mL (*P* values = 0.0). The fold change in caspases 3/7 activity was 3.2 ± 0.7 and 1.3 ± 0.1 in the 2.5/5.0 and 2.5/7.5 α-mangostin/BA combinations, respectively, and the fold change in the 3.75/5.0 and 3.75/7.5 combinations was 8.0 ± 2.3 and 2.8 ± 0.2, respectively.

### 2.5. Effect on Cellular Morphology, Mitochondrial Membrane Potential and Chromatin Condensation

**Figure 6:**
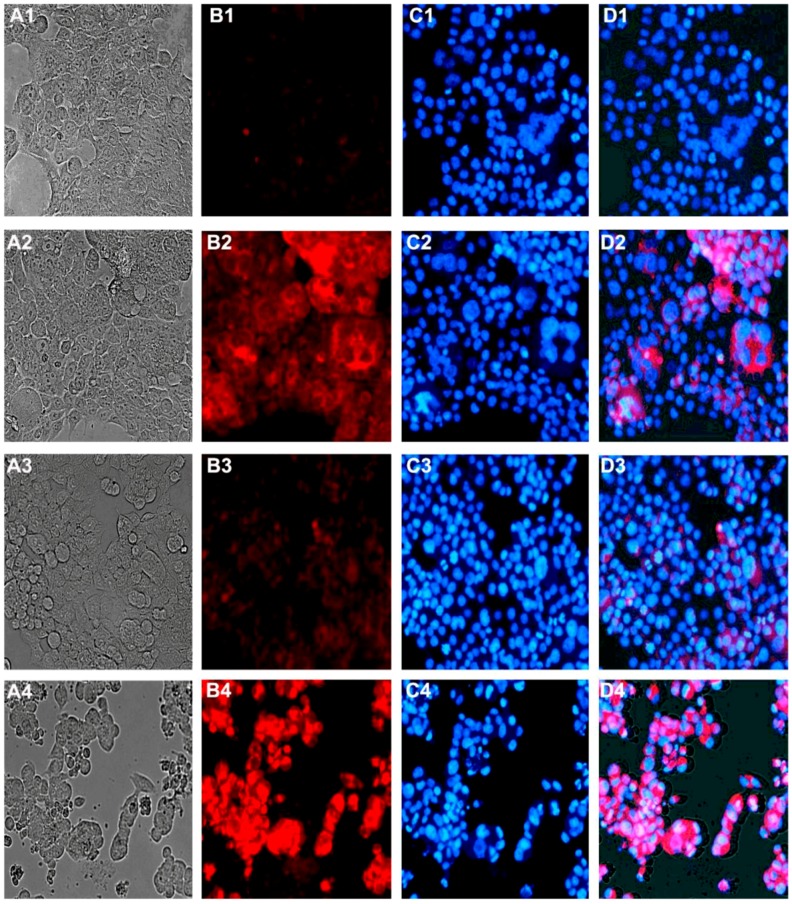
Effect of α-mangostin, betulinic acid (BA) and their combination on the morphology, mitochondrial membrane potential, and chromatin condensation of HCT 116 cells. Untreated cells (**A1–D1**), treated with BA at 7.5 µg/mL (**A2–D2**), treated with α-mangostin at 3.75 µg/mL (**A3–D3**), and their combination (**A4–D4**). The treatment time was 16 h, and (**1**) refers to light micrograph of unstained cells; (**2**) stained with rhodamine 123, (**3**) stained with Hoechst 33258, and (**4**) is an overlay of the 3 micrographs.

**Figure 7:**
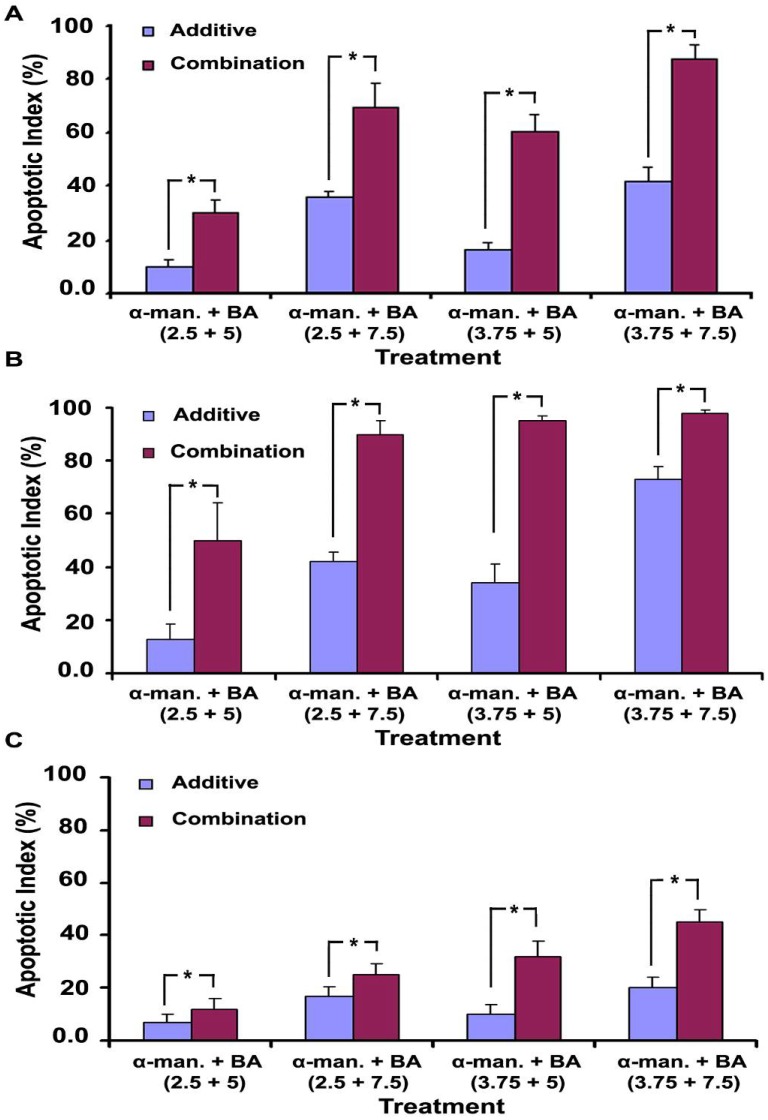
Apoptotic index of the combinations of α-mangostin and betulinic acid (BA) on HCT 116 cells. The effect on cellular morphology (**A**), the loss of mitochondrial membrane potential (**B**), and the chromatin condensation (**C**). The combination effect was compared to the additive effect by student’s *t*-test, and (*) refers to *P* values < 0.05.

α-Mangostin, BA and their combinations induced apoptotic morphological changes of HCT 116 cells including cytoplasmic shrinkage, loss of attachment, and the formation of apoptotic bodies [Figure 6(A1–A4)]. The apoptotic index was determined, and the combination effect was compared to the additive effect of the individual treatments. Statistical analysis by student’s t-test showed that the apoptotic index of α-mangostin/BA combinations is significantly higher than their additive effect, *P* < 0.05 (Figure 7A). The highest apoptotic index was 92 ± 5%, which was achieved in α-mangostin/BA combination (3.75/7.5 µg/mL). 

Staining with rhodamine 123 showed a distinct morphology of the apoptotic cells, which appeared brighter than their non-apoptotic counterparts [Figure 6(B1–B4)]. Rhodamine 123 accumulates in the mitochondria of viable cells where it forms dye aggregates leading quenching of its fluorescence especially at high concentration [23], which may explain the reduced fluorescence intensity observed in the untreated cells (viable cells). On the other hand, the loss of mitochondrial membrane potential in apoptotic cells is associated with efflux of rhodamine 123 from the mitochondria into the cytosol leading to de-quenching of the dye and ultimately increasing the overall cellular fluorescence intensity [24]. Hence, the increased cellular fluorescence observed in the treated cells most likely occurred due to the loss of mitochondrial membrane potential. The increase in rhodamine fluorescence in dead cells was previously reported by other researchers [25]. Apoptotic index data indicate substantial improvement in the apoptotic effect of α-mangostin/BA combinations over their additive effect, *P* < 0.05 (Figure 7B). 

α-Mangostin (2.5 and 3.75 µg/mL), or BA (5.0 and 7.5 µg/mL) alone caused minor changes on the nuclear morphology of HCT 116 cells. On the other hand, the combination of both compounds induced characteristic changes in the chromatin structure and nuclear morphology of the cells. Chromatin condensation with smaller nuclei, half moon or crescent-shaped nuclei, nuclear fragmentation, and formation of apoptotic bodies were all observed [Figure 6(C1–C4)]. The nuclear changes induced by the combination were significantly higher than the individual treatments and their additive effect. The highest apoptotic index was 47 ± 5%, which was obtained in α-mangostin/BA combination (3.75/7.5 µg/mL) (Figure 7C). However, the percentage of apoptotic cells showing nuclear changes was less than those showing loss of mitochondrial potential and cytoplasmic shrinkage.

### 2.6. Effect on Cell Signaling Pathways

In order to get deeper insights into the mechanism of action of α-mangostin, its effects on four cell signaling pathways was investigated by the luciferase assay. α-Mangostin at a concentration of 7.5 µg/mL caused significant enhancement of the p53/DNA damage (1.4 ± 0.2 folds), Myc/Max (1.6 ± 0.1 folds), and the MAPK/ERK (2.1 ± 0.4 folds) signaling pathways, and downregulation of the NFKB pathway (0.8 ± 0.1 folds), *P* values < 0.05.

## 3. Discussion 

α-Mangostin and BA are cytotoxic compounds of natural origin. In this study α-mangostin was isolated at high purity from a xanthone extract from *Garcinia mangostana* Linn, and BA was isolated from a BA fraction from *Syzygium campanulatum* Korth. The identity of the compounds was confirmed by comparing their HPLC retention time and MS isotopic pattern with reference compounds. Both compounds have been reported to have anti-cancer activity, which is mediated via the mitochondrial apoptosis pathway [5,26]. Previous reports indicated that compounds that work directly on the mitochondria may evade resistance to conventional chemotherapeutics [15], consequently BA and α-mangostin may hold a great promise as a novel approach to overcome drug resistance in cancer chemotherapy.

This study aimed to study the cytotoxic effect of α-mangostin/BA, α-mangostin/cisplatin, and BA/cisplatin combinations on colorectal carcinoma cells. HCT 116 human colorectal carcinoma cell line was selected because it was reported as an invasive and metastatic model of colorectal carcinoma [27], and CCD-18Co was selected as a control normal cell line. The compounds showed a potent and dose dependent cytotoxicity towards the HCT 116 human colorectal carcinoma cells, which was 2.8-fold (α-mangostin) and 4.7-fold (BA) less cytotoxic on the normal colonic cells. However, their combinations showed different cytotoxicity profiles; at sub-cytotoxic concentrations, the compounds significantly inhibited cisplatin cytotoxicity. Whereas, cytotoxic concentration of both α-mangostin and BA did not show the same cytoprotective effect, but resulted in almost 100% cell killing. 

Previous reports showed that the DNA damaging agents such as cisplatin induce apoptosis through the accumulation of reactive oxygen species (ROS) [28]. α-Mangostin has been reported with ROS scavenging capacity [29], and recent reports showed a cytoprotective effect of α-mangostin against cisplatin-induced cytotoxicity on renal tubular cells, which was due to the inhibition of ROS generation [30]. Likewise, BA has been reported to possess a vascular protective effect against tumor necrosis factor-alpha (TNF-α) induced inflammation [31], and a cytoprotective effect against acetaminophen and ethanol-induced cytotoxicity on HepG2 cells [32]. In both reports the authors concluded that the cytoprotective effect of BA was due to inhibition of ROS generation. Consequently, the cytoprotective effect of α-mangostin and BA on cisplatin-induced cytotoxicity of colon cancer cells may be due to the inhibition of ROS generation. It seems that the ROS scavenging effect of BA and α-mangostin is more evident at low concentrations, which suggests dual effects of the compounds; apoptotic effect at high concentration and cytoprotective effect at low concentration. The data presented in this study and the results of other research groups provide experimental evidence on the *in vitro* cytoprotective effect of α-mangostin and BA against cisplatin and other cytotoxic compounds on cancer and normal cells. Further investigation is needed in order to study their *in vitro* and *in vivo* cytoprotective effects on other cytotoxic compounds against other normal and cancer cell lines, and to study their mechanism of action. 

The α-mangostin/BA combination resulted in enhancement of their cytotoxic effect towards the colon cancer cells but not towards the normal colonic cells. This effect was further investigated on the early and late markers of apoptosis including cytoplasmic shrinkage and loss of attachment, enhancement of caspases-3/7 activity, loss of mitochondrial membrane potential, and chromatin condensation and nuclear morphology. Our data showed that the apoptotic effect α-mangostin/BA combination is significantly higher than their additive effect, which confirm the enhancement effect of the combination. It is noteworthy to mention that the effect on the mitochondrial membrane potential was stronger than that on chromatin condensation and nuclear morphology, which may indicate the direct effect of the combination on the mitochondria. We found that the apoptotic effects of α-mangostin are mediated via upregulation of p53, Myc/Max and MAPK/ERK signaling pathways and downregulation of the NFKB pathway. BA has been shown to work by inhibition of the hypoxia pathway [33], decreasing expression of the anti-apoptotic protein Bcl2 and cyclin D1 [34], and increasing the expression of the pro-apoptotic proteins Bax and caspase 3 [35]. Since BA and α-mangostin target different signaling pathways, the compounds may act synergistically when used in combination. Besides the enhancement of BA cytotoxicity by α-mangostin, this combination may provide other advantages such as decreasing the likelihood of developing drug resistance, and the anti-angiogenic effect of BA [7,33] may inhibit tumor metastasis by inhibiting the tumor angiogenesis.

## 4. Experimental

### 4.1. Cell Culture and Reagents

Human colorectal carcinoma cell line HCT 116, and human normal colonic cell line CCD-18Co were purchased from the American Type Culture Collection (ATCC; Manassas, VA, USA). RPMI 1640, Opti-MEM^®^ and DMEM cell culture media, heat inactivated fetal bovine serum (HI-FBS), and phosphate buffered saline (PBS) without calcium and magnesium were purchased from Bio-Diagnostics (Petaling Jaya, Selangor, Malaysia). The Cignal finder™ reporter-array system was purchased from SABiosciences (Frederick, MD, USA). Caspases-3/7 reagent, and trans-fast liposome, and dual luciferase reporter system were purchased from Promega (Petaling Jaya, Selangor, Malaysia). Cisplatin, BA, Hoechst 33258, rhodamine 123, penicillin/streptomycin (PS), 2,3-bis(2-methoxy-4-nitro-5-sulfophenyl)-2*H*-tetrazolium-5-carboxanilide inner salt (XTT), and phenazine methosulfate (PMS) were purchased from Sigma-Aldrich (Kuala Lumpur, Malaysia). α-Mangostin reference compound was purchased from ChromaDex (Irvine, CA, USA). 

### 4.2. Isolation and Characterization of BA

BA was purified from a BA fraction that was prepared from *Syzygium campanulatum* leaf extract as previously described [36]. The column (25 × 1 inch) was packed with 50 g silica gel 60 (0.063–0.200 mm, Merck). The BA fraction (1 g) was dissolved in 1:1 methanol-chloroform (10 mL), mixed with silica gel (5 g), and the dried mixture was then loaded into the pre-packed column. Elution was performed with *n*-hexane-ethyl acetate at 8:2 ratio (v/v), and 10 mL fractions were collected. The purity of BA was determined by HPLC as previously described [36]. Briefly, the column was a ZORBAX Eclipse Plus C18 (5 µm, 4.6 × 250 mm), the mobile phase was made up of 86% acetonitrile and 14% 0.1% H_3_PO_4_ in water, the flow rate was set at 1 mL/min for 20 min, and the spectral data was collected at 210, 230 and 254 nm.

### 4.3. Isolation and Characterization of α-Mangostin

α-Mangostin was isolated by column chromatography from a toluene extract prepared from *Garcinia mangostana* fruit rinds. The column (25 × 1 inch) was packed with 60 g silica gel 60 (0.063–0.200 mm, Merck). The xanthone extract (1 g) was dissolved in methanol and mixed with silica (5 g), and the dried mixture was loaded into the pre-packed column. Elution was then performed with 200 mL portions dichloromethane-*n*-hexane in 1:3, 1:2 and 2:1 ratios (v/v). Methanol was then added at 0.5% (v/v) with a two fold increment in each 200 mL cycle to reach 4% in the fourth one. Ten millilitre fractions were collected, and the purity of α-mangostin was tested using Dionex-Ultimate® 3000 Rapid Separation HPLC. The column was Nucleosil C18 (5 µm, 4.6 × 250 mm), the column temperature was set at 30 °C, the mobile phase was 95% acetonitrile:5% 0.1% H_3_PO_4_ in water for 10 min at 1 mL/min, and data was collected at 244, 254, 316 and 320 nm (submitted manuscript). 

### 4.4. Direct-Infusion Electrospray Mass Spectrometry

The identity of the isolated compounds was further confirmed by MS analysis. MS spectra of α-mangostin and BA were obtained using a LC-MSD Trap-VL Electrospray ion (ESI) mass spectrometer (Agilent Technologies) in the direct infusion mode. The samples were injected directly into the ESI source at 5 μL/min. The MS conditions were as follows: negative ion mode; gas (N2) temperature, 325 °C; flow rate, 5.0 L/min; nebulizer pressure, 15 psi; HV voltage, 4.0 kV; octopole RF amplitude, 150 Vpp; skim 1 voltage, −38.8 V; skim 2 voltage, −6.0 V; capillary exit, −113.8 V; cap exit offset, −75.0 V; scanning range, 350–550 *m/z*, and the acquired mass spectra represent the average of five spectra.

### 4.5. Cell Viability

HCT 116 cells were propagated in RPMI 1640 medium supplemented with 10% HI-FBS and 1% PS. CCD-18Co cells were maintained in DMEM medium with 10% HI-FBS and 1% PS. Cells were cultured at 37 °C in a 5% CO_2_ humidified atmosphere. Cell viability was determined by the XTT test as described previously [37]. Briefly, cells were treated with the individual compounds and their combinations for 48 h, the culture medium was removed and replaced with a fresh one containing XTT and PMS at 100 µg/mL, and 1 µg/mL, respectively. After 4 h incubation, the optical density was measured at 450 nm using a microplate reader (Hitachi U-2000, Japan). The results are presented as either percentage viability or percentage inhibition to the negative control (0.5% DMSO), and the IC_50s_ were calculated from the dose response curves (n = 3).

### 4.6. Caspases-3/7

The caspases 3/7 activity was measured by caspase Glo 3/7 as described previously [38]. In brief, HCT 116 cells were treated for 16 h with α-mangostin, BA, or their combinations. Subsequently, caspase 3/7 reagent was added at 1:1 ratio (v/v), and luminescence was measured after 30 min incubation using a microplate reader. The results are presented as a mean of relative light units (RLU) ± SD (n = 4). 

### 4.7. Mitochondrial Membrane Potential and Chromatin Condensation 

Rhodamine 123 and Hoechst 33258 were used as probes to study the effect on mitochondrial membrane potential and chromatin condensation [39,40]. Briefly, HCT 116 cells were treated with α-mangostin, BA, or their combinations for 16 h. Subsequently, cells were fixed in 4% paraformaldehyde for 20 min, simultaneously stained with rhodamine 123 at 1 µg/mL and Hoechst 33258 at 10 µg/mL for 20 min, washed extensively with PBS, and examined immediately under the red filter (rhodamine 123) and the blue filter (Hoechst 33258) of a EVOS^®^ digital fluorescent inverted microscope (Advanced Microscopy Group, Bothell, WA, USA). Cell morphology was evaluated by studying five randomly selected microscopic fields, and the apoptotic index was calculated. 

### 4.8. Luciferase Assay

The effect of α-mangostin on four cell signaling pathways was investigated by the luciferase assay as described previously [41]. In brief, HCT 116 cells were transfected with the DNA constructs of four signaling pathways (p53/DNA damage, NFKB, Myc/Max, and MAPK/ERK) by reverse transfection using trans-fast liposome transfection reagent. After overnight incubation, cells were treated for 6 h, and the activity of the transcription factors was measured using Firefly/*Renilla* dual-luciferase assay according to manufacturer’s instructions. The Firefly/*Renilla* ratio was then calculated, and the fold change in the transcription factor activity was determined by dividing the results of the treated cells by that of untreated cells (0.5% DMSO).

### 4.9. Statistical Analysis

The results are presented as mean ± SD. The additive effect of the individual treatments was calculated as described previously by Webb, the following equation was used [42]:

Additive effect = 1 − A

where A = (1 − x) × (1 − y), where x and y are the effects of the individual treatments. The differences between groups were compared by student’s *t*-test or One-way ANOVA, and were considered significant at *P <* 0.05. Data analysis was carried out using SSPS 16.0 software.

## 5. Conclusions

The cytoprotective effect of BA and α-mangostin against cisplatin induced cytotoxicity may indicate a dual effect of these compounds in cancer chemotherapy; they may neutralize the nonselective cytotoxicity of cisplatin or other chemotherapeutics, or may reduce their overall anti-cancer effect, which needs further *in vivo* investigation. The compounds may also provide a cytoprotective effect against oxidative stress, irradiation, and chemical carcinogens, which may benefit in prevention of several human diseases such as cancer, neurodegenerative disorders, aging, and cardiovascular diseases. 

The enhancement of BA cytotoxic and apoptotic effects by α-mangostin may indicate this combination as a novel candidate for the treatment of colorectal carcinoma.

## Figures and Tables

**Table 1:** Summary of the cytotoxic effect of α-mangostin, BA, and cisplatin and their combinations on HCT 116 cells. The table presents the percentage inhibition of individual treatments and their combinations, and the fold change in the cytotoxicity of combinations compared to their additive cytotoxicity. The results are displayed as average ± SD (n = 3).

**1-α-Mangostin (2.5 µg/mL) and betulinic acid (BA)**					
**BA** **(µg/mL)**	**BA** **(% inhibition)**	**α-Man.** **(% inhibition)**	**Combination** **(% inhibition)**	**Additive** **(% inhibition)**	**Fold change in BA cytotoxicity**
**2.5**	4 ± 0.2	2.0 ± 1.7	51 ± 2.5	6.5 ± 1.1	7.9 ± 0.9
**5.0**	9 ± 1.0		91 ± 1.6	11.6 ± 1.5	7.9 ± 0.8
**7.5**	20 ± 1.5		99 ± 0.6	22.3 ± 1.8	4.5 ± 0.4
**10.0**	50 ± 2.0		100 ± 0.1	51.4 ± 2.1	1.9 ± 0.1
**2-α-Mangostin (2.5 µg/mL) and cisplatin (Cis.)**					
**Cis.** **(µg/mL)**	**Cis.** **(% inhibition)**	**α-Man.** **(% inhibition)**	**Combination** **(%inhibition)**	**Additive** **(% inhibition)**	**Fold change in Cis. cytotoxicity**
**2.5**	16 ± 2.3	2 ± 1.7	18 ± 3.3	18.8 ± 2.2	1.0 ± 0.09
**5.0**	36 ± 1.5		12 ± 3.6	37.9 ±1.7	0.3 ± 0.08
**7.5**	83 ± 1.0		9 ± 0.3	83.5 ±1.0	0.1 ± 0.01
**10.0**	97 ± 1.0		19 ± 4.5	97.1 ±1.0	0.2 ± 0.04
**3-BA (2.5 µg/mL) and Cisplatin.**					
**Cis.** **(µg/mL)**	**Cis.** **(% inhibition)**	**BA** **(% inhibition)**	**Combination** **(% inhibition)**	**Additive** **(% inhibition)**	**Fold change in Cis. cytotoxicity**
**2.5**	16 ± 2.3	4.0 ± 0.2	18 ± 1.2	19.7 ± 1.9	0.9 ± 0.03
**5.0**	36 ± 1.5		28 ± 0.5	38.6 ± 1.6	0.7 ± 0.02
**7.5**	83 ± 1.0		49 ± 1.8	83.7 ±1.0	0.6 ± 0.01
**10.0**	97 ± 1.0		72 ± 1.2	97.1 ±1.0	0.7 ± 0.01
